# Differential expression of long non-coding RNAs as diagnostic markers for lung cancer and other malignant tumors

**DOI:** 10.18632/aging.203523

**Published:** 2021-10-20

**Authors:** Li Li, Haitao Wei, Yi Wei Zhang, Shizhe Zhao, Guowei Che, Yun Wang, Longqi Chen

**Affiliations:** 1 College of Nursing and Health, Henan University, Kaifeng, Henan 475004, China; 2 Department of Thoracic Surgery, West China Hospital, Sichuan University, Chengdu, Sichuan 610041, China; 3 Department of Thoracic Surgery, Huaihe Hospital, Henan University, Kaifeng, Henan 475001, China; 4 Basic Medical College of Henan University, Kaifeng, Henan 475004, China

**Keywords:** long non-coding RNAs, lung cancer, differential expression, diagnostic markers, clinical application

## Abstract

Due to advances in chip and sequencing technology, several types and numbers of long non-coding RNAs (lncRNAs) have been identified. LncRNAs are defined as non-protein-coding RNA molecules longer than 200 nucleotides, and are now thought as a new frontier in the study of human malignant diseases including NSCLC. Diagnosis of numerous malignant tumors has been closely linked to the differential expression of certain lncRNAs. LncRNAs are involved in gene expression regulation at multiple levels of epigenetics, transcriptional regulation, and post-transcriptional regulation. Mutations, deletions, or abnormal expression levels lead to physiological abnormalities, disease occurrence and are closely associated with human tumor diseases. LncRNAs play a crucial role in cancerous processes as either oncogenes or tumor suppressor genes. The expression of lncRNAs can regulate tumor cell in the proliferation, migration, apoptosis, cycle, invasion, and metastasis. As such, lncRNAs are potential diagnostic and treatment targets for cancer. And that, tumor biomarkers need to be detectable in easily accessible body samples, should be characterized by high specificity and sufficient sensitivity. Herein, it is significant clinical importance to screen and supplement new biomarkers for early diagnosis of lung cancer. This study aimed at systematically describing lncRNAs from five aspects based on recent studies: concepts, classification, structure, molecular mechanism, signal pathway, as well as review lncRNA implications in malignant tumor.

## INTRODUCTION

Globally, lung cancer is a malignant tumor with the highest mortality rates. Lung cancer associated mortalities in China account for one-third of the global lung cancer mortality [1]. At the time of clinical diagnosis, many lung cancer patients are already at a locally advanced or metastatic stages, resulting in a poor disease prognosis. The diagnosis, treatment, and prognosis of lung cancer is inhibited by various challenges. Elucidating the molecular mechanisms associated with carcinogenesis can help reveal specific cancer biomarkers that can be utilized in personalized therapy. Clinically, lung cancer is divided into two forms: small-cell lung cancer (SCLC) and non-small cell lung cancer (NSCLC), with adenocarcinoma being the most common subtype in China. LncRNAs studies can unravel the multi-level expression regulation network in living organisms, and provide new bio-markers for targeted therapy as well as diagnostic prognostic, and treatment targets. Although the role of several lncRNAs in tumorigenesis has been elucidated, the molecular biological mechanism of lncRNAs in lung cancer pathogenesis is poorly studied. The Human Genome Project revealed that only 1–2% of the genome of cancer cells encodes proteins, whereas the rest of 98% of the genome generate non-coding RNAs (ncRNA) [2]. Approximately two-thirds of human genome does not encode protein, and many are intergenic with most coding for long (>200 nucleotides) non-coding RNAs (lncRNAs). Although lncRNAs lack a protein-coding ability, they are directly involved in a regulatory role at the RNA level, including growth and development, bone marrow hematopoiesis, apoptosis, and cell proliferation [3]. These transcriptional non-coding sequences are widely involved in human physiological and pathological activities and even tumor progression, increasing the complexity of eukaryotes [4]. We initially hypothesized that the majority of ncRNAs could be transcriptional noises and mere by-products of the transcription of RNA polymerase II without biological functions [5]. However, recent developments have revealed the critical role ncRNAs play in cell proliferation, cycle, apoptosis, invasion, and metastasis. Regardless to whether the fragment is longer than 200 nucleotides or less, ncRNAs can be classified into two groups: small non-coding RNAs are less than 200 nt, whereas lncRNAs are longer than 200 nt. Several small non-coding RNAs have been identified as potential markers for tumor-targeted chemotherapy. LncRNAs are involved in the gene life cycle process and are closely associated with the pathogenesis of many complex diseases. As such, they have been identified as potential disease biomarkers and drug target. The Encode project identified nearly 16,000 lncRNAs, demonstrating the diversity of lncRNAs [6]. However, only a few hundred lncRNAs have been reported to have specific functions. Besides regulating gene expression and the biological mechanisms underlying tumorigenesis, lncRNAs influence prognosis of NSCLC. Therefore, this study evaluated the role of lncRNAs in diagnosis, prognosis and treatment of NSCLC.

## The classification of lncRNAs

LncRNAs play an important role in various biological processes. Based on their biological functions, ncRNAs with limited or no protein-coding ability are divided into housekeeping ncRNAs and regulatory ncRNAs. Housekeeping RNA is necessary for cell survival and is always present in cells in constant levels. It is less affected by environs, and is essential for maintaining cell function. They mainly include tRNA, rRNA, snRNA, snoRNA and telomerase RNA, etc. Regulatory ncRNAs are ubiquitously expressed in a spatial and/or temporal specific pattern. Moreover, regulatory ncRNAs can be further classified into two subcategories based on their size: small ncRNAs (sncRNAs) and lncRNAs. Short non-coding RNAs including miRNAs, siRNAs, and piRNAs are less than 200 nt long. Long non-coding RNAs and bigger than 200 nt, do not have long open reading frames, and do not code for proteins. Under specific conditions, certain lncRNAs code for micropeptides. LncRNAs constitute 70% of ncRNAs and regulate gene expression via a several mechanisms. Poning et al. divided lncRNAs into 5 types according to their positions in the genome relative to protein-coding genes: (1) Sense lncRNAs, located in the same strand of a protein-coding gene; (2) Antisense lncRNAs, when overlapping one or more exons of other transcript on the same, or opposite, strand, respectively; (3) Bidirectional lncRNAs, when the expression of it and a neighboring coding transcript on the opposite strand is initiated in close genomic proximity; (4) Intronic lncRNAs, when it is derived wholly from within an intron of a second transcript (although these, as noted above, sometimes may represent pre-mRNA sequences), or (5) Intergenic lncRNAs (lincRNAs), located between protein coding regions, when it is derived wholly from within an intron of a second transcript (although these, as noted above, sometimes may represent pre-mRNA sequences) [7]. Based on subcellular localization, lncRNAs can also be classified into two classes: (1) nuclear lncRNAs, are involved in gene transcription and chromatin remodeling, and (2) cytoplasmic lncRNAs, typically regulate RNA-mediated functions [8]. Recent research, however, has shown that lncRNA participate in the processes of silencing X chromosome, chromosome modification, and genome modification, transcription activation and interference, and intranuclear transport, etc. [9]. Given that lncRNAs are expressed cell- and tissue-specific, they are excellent biomarkers for ongoing biological events [10]. The role of lncRNAs in tumor development continues to receive significant attention.

## Structure of lncRNAs

LncRNAs can be spliced, capped, and/or polyadenylated molecules widely distributed in the nucleus or cytoplasm of eukaryotes. LncRNAs are mainly transcribed by RNA polymerase II, and structurally similar to mRNA, equipped with a poly (A) tail and promoter regions. LncRNAs also often display a secondary stem-loop-like structures or tertiary structures, occasioned by base complementation. The secondary structure consists of a helix, hairpin, bulge and pseudoknot, formed by complementary base pairing. In many cases, the structure, rather than the original sequence, determines the RNA's function. Depending on its base specificity or structure specificity, lncRNAs strongly regulate gene expression by interacting with nucleic acids or proteins. LncRNAs are tissue-specific and space-time specific and interact with RNA, DNA, and protein to regulate gene expression at multiple levels, including epigenetically, transcriptionally, post-transcriptionally and through splicing. The majority of the lncRNAs are polyadenylated in the nucleus or cytoplasm, showing a strong ability to regulate gene expression based on their base specificity or structure specificity in interacting with nucleic acids or proteins [11]. LncRNAs can form more complex structures than protein-coding genes, and their structural diversity enables them to participate in establishing binding sites that interact with proteins, DNA, and other RNA molecules. This implies that the structure of lncRNAs is closely related to their biological function. Nevertheless, the study of the lncRNA folding process, including the search for cofactors and the relationship between non-coding structures and functions, is still in the initial stage and it could be an important step for future studies in revealing the regulatory mechanism of lncRNAs [12].

### Primary structure of lncRNAs

The original structure of the lncRNAs is composed of the nucleotide array with 5-methylguanosine cap structure and a 3-polyadenylation tail structure. To perform their regulatory functions, lncRNAs must be located at special positions in the nucleus. They regulate gene function in several ways, the most importantly through direct interaction with target gene and post transcriptionally by complementary base pairing with target genes or regulating genes transcriptional translation upstream or downstream of the target gene. Base pairing is based on its primary structure. It has been reported that lncRNA Gas5 can directly bind to the glucocorticoid DNA binding domain, then compete with the target genes containing the glucocorticoid response element, thus and regulating its expression [13]. Complementary pairing with the target bases is based on the primary structure of lncRNA. The lncRNAs either target miRNA or mRNA. For example, Linc-MD1 binds miR133 and miR135 through base complementary pairing, competitively inhibiting interaction of the two miRNAs with target genes [14]. LncRNAs termed 1/2sbsRNAs can incompletely pair with the Alu element in the 3'-UTR region of the mRNA to form binding site of the RNA binding protein Stau1. This promotes the binding of Stau1 to mRNA, and degrading mRNA through the stau1-mediated mRNA decay (SMD) pathway [15].

### Secondary structure of lncRNAs

The secondary structure of lncRNAs is the center of lncRNA function. In 2012, a study reported the secondary structure information of lncRNA steroid receptor RNA activator (SRA). LncRNA SRA can activate expression of several sex hormones receptors and is closely related to the development of breast cancer [16]. Some studies indicate that secondary structure may be the key to lncRNA function. For instance, MALAT1, a highly conserved uracil-rich region, increases RNA stability by forming a triple helix [17]. The study of two secondary collapsible motifs can contribute to the anticancer effect of LncRNA MEG3 [18]. Novikova et al. found that due to compensatory mutation, the specific folding of SRA can be preserved in the human species [19]. In conclusion, there is increasing evidence that the structure of lncRNAs is important to its function. However, the sequence evolution of lncRNAs with low effective population size remains to be determined within the whole genome sequences regardless of the schema of secondary structure.

## Molecular mechanism of lncRNAs

LncRNA is increasingly recognized as a major regulator of gene expression, existing at almost all levels of the gene's life cycle, including chromatin modification, transcription, and post-transcriptional processing. The biological function of lncRNAs is strictly dependent on its cellular location. LncRNAs are usually responsible for chromatin modification, transcription regulation, and mRNA or miRNA processing in the cell nucleus. In the cytoplasm, lncRNAs regulate translation, or act as miRNA. LncRNAs also act as molecular sponges, where they alter miRNA levels and accelerate or suppress the transcription of key genes, including albumen genes [20, 21]. Unlike highly conserved sncRNAs, lncRNAs regulate expression of target genes via various mechanisms [22, 23]. LncRNAs are a class of ubiquitous genes that are not only involved in the normal biological functions of the body but also multiple processes of disease. LncRNAs realize their molecular functions through signals, decoy, guides, and scaffold: ①The Signal lncRNAs can be used as markers in many biological processes because they display cell type, time and interspace specificity. As signaling molecules, lncRNAs regulate numerous signaling pathways. Some lncRNAs regulate the transcription of downstream genes and can reflect the temporal and spatial expression of genes. For example, the lncRNA homeobox (HOX) transcriptional antisense inter-gene RNA (HOTAIR) located at the HOXC site is present in the posterior and distal cells, while the other lncRNA HOXC are expressed in the fore-end pattern [24]. In contrast, the lncRNA HOXA transcript is located in distal cells [25]. ②The Decoy lncRNAs are those that adjust transcription by combining and carrying protein targets but do not cause other effects. The decoy lncRNAs which are known as “molecular sensors”, reduce the availability of regulatory factors including catalytic proteins, subunits of chromatin modifying complexes, transcription factors, and miRNAs by presenting binding sites. The decoy lncRNAs include p21-associated non-coding RNAs (PANDA), non-coding RNAs containing telomere repeats (TERRA), miRNAs, and molecular baits splicing silver [26]. For example, by immediately combining nuclear transcription factor Y that forces apoptosis induced by DNA injury, activated lncRNA p21 damage inhibits apoptotic gene, promoting progression of the cell cycle [27]. ③The Guide lncRNAs can induce chromatin decorating proteins and administration of ribonucleoprotein complexes to specific targets in a cis or trans manner. The famous cis mechanism involves the mammalian X-inactivation center (XIC), and X-inactive-specific transcript (XIST). A 1.6-kb ncRNA, (RepA) recruits poly comb repressive complex 2 (PRC2) in cis on the Xist, as its direct target. PRC2 is associated with extra X-chromosome inactivation [28]. LncRNAs influence their chromosome-wide transcription in trans unlike in cis-regulated lncRNAs. The lncRNA HOTAIR, for instance, is equipped to guide PRC2 target genes [29]. ④The scaffold lncRNAs can be used as a terrace for packaging components to accurately regulate the complex molecular interactions and signal transductions involved in several biological signaling processes [30]. The telomerase catalytic process, for example, requires telomerase RNA (TERC) and telomerase reverse transcriptase (TERT) attachment. TERC is a fundamental lncRNA unit that initiates the combination template, and also reveals the intricate territories that promote TERT banding, catalytic activity, and stability [31]. Dyskeratosis is a congenital disorder caused by mutations that alter the equilibrium between various TERC structures, due to the destruction of the structure of the RNA scaffold where telomeric regulatory protein modular binding sites are located [32].

### LncRNAs mediate epigenetic regulation

**Figure 1:**
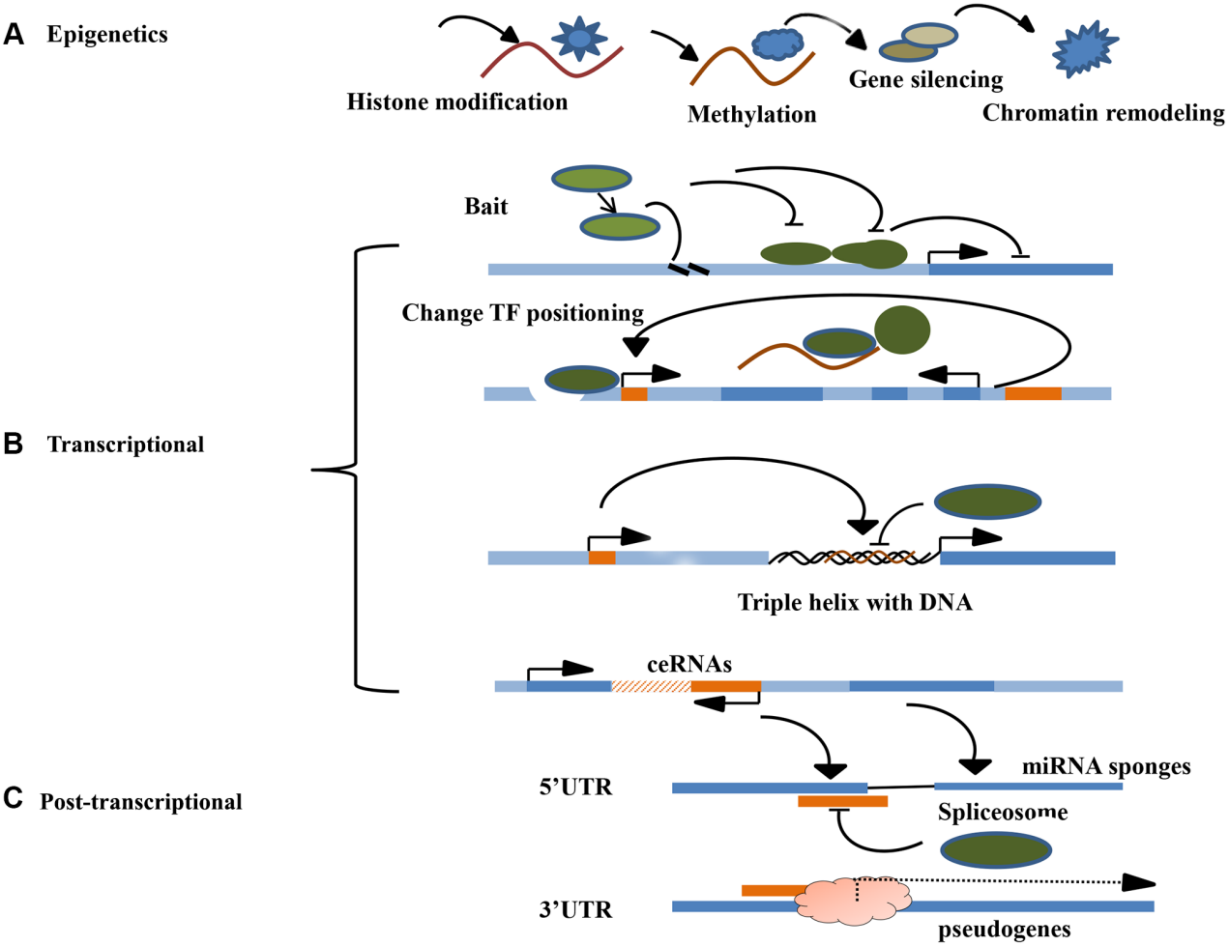
**Molecular mechanism of LncRNA.** (**A**) The main mechanisms of lncRNA epigenetics include histone modification, DNA methylation, gene silencing, and chromatin remodeling. (**B**) The main if lncRNA transcription level include signal induction, regulation of transcription factor sites, DNA triple helix structure and co-expression of ceRNA. (**C**) The main mechanisms of lncRNA post-transcriptional level include guiding the selective splicing of miRNA subtypes and targeting the protein receptor complex to recognize righteous chain mRNA transcripts.

Epigenetics refer to genetic alterations in gene expression without changes in the underlying DNA sequences. They include DNA methylation, chromatin remodeling, histone modification, and gene-related non-coding RNA silencing [33]. The most important function of lncRNAs is to regulate the epigenetic modification of the target gene. During embryonic development, epigenetic modification regulate several important life activities processes, such as genomic imprinting, chromosomal silencing, and chromosomal dosage compensation effects, all necessary for the normal embryonic development and differentiation of tissue cells [34]. Histones can be regulated and modified in various ways, including methylation, acetylation, and ubiquitination. The modifications affect gene expression differently. Regulatory methylation modification is the major form of epigenetic regulation, and affects the expression of related genes by regulating the CpG island methylation level in the gene promoter region. Chromatin remodeling studies have shown that epigenetic regulation can be combined with chromatin modification complexes to regulate chromatin remodeling in human species genes, influence gene expression, and participate in various life processes *in vivo*, such as the development of diseases, including tumors [35]. Currently, there are no accurate methods for predicting the pathogenesis and prognosis of NSCLC. Epigenetic gene expression regulation mechanisms also play an important role in tumorigenesis. Besides genetic changes in DNA sequences, several epigenetic changes in tumors diversify gene expression diversification and signaling pathways in malignant cells [36]. Studies have shown that lncRNAs can regulate the progression of lung cancer [37]. They initiate epigenetic changes that induce chromosomal modification complexes to specific genomic sites. Chromosomal instability is an important factor associated with tumor progression and metastasis. Overall, lncRNAs can function as oncogenes and tumor suppressor genes. Increasing evidences has implicated that lncRNA in the occurrence and development of NSCLC [38]. However, in the role of lncRNA in the prognosis of lung cancer has not been established (Figure 1A).

### LncRNAs mediate transcriptional regulation

Regulation of adjacent mRNA transcriptional expression by lncRNAs is common in eukaryotes. Several lncRNAs combine with suitable transcription factors to regulate their transcriptional activity. Studies show that certain lncRNAs can act as receptors to modulate base-pairing interchanges for these transcription-related elements, thus guiding the lncRNA including complex targeting spots for specific RNA or DNA. By acting as transcription regulatory factors, lncRNAs can regulate the transcription of cis-acting elements (adjacent genes) or trans-acting elements (distal genes) positively or negatively as transcription regulatory factors. The specific processes are: ①As decoys for RNA polymerase II or transcription factors (TFs), inhibiting its binding to the enhancer could become the promoter of the target gene, thereby, directly promoting or inhibiting the target gene manifestation [39]; ②Changing the location or modification of TF to accelerate or suppress gene transcription; ③Interacting with DNA to form a triple helix structure to mediate target gene transcription; ④Controlling target gene transcription in the form of competitive endogenous RNAs(ceRNAs). Studies have shown that lncRNAs can silence miRNA expression to promote tumor progression. Over-expression of LncRNA HOTAIR promotes transformation of malignant tumors, leading to a poor prognosis [40]. Furthermore, HOTAIR combines with EZH2 and SUZ12, and directly binds miR-34a promoter region, regulating expression level of miRNA [41]. Suppressing miR-34a expression activates the PI3K/AKT signaling that enhances lung cancer development [42]. LncRNAs can directly regulate gene expression by binding transcription factors and/or polymerase II, driving them away from or guiding them to target genes sites; or indirectly affecting gene expression by regulating transcription localization of proteins, such as promoting or inhibiting their nuclear localization (Figure 1B).

### LncRNAs mediate post-transcriptional regulation of gene expression

lncRNAs regulates gene expression at the post-transcriptional level through manifold mechanisms, including mRNA compilation and commutative joint, and serves as a storage for sncRNAs and miRNA sponge layers. RNA compilation is a significant post-transcriptional process that embellishes, inserts, or deletes nucleotides to change the RNA molecule. The most common editing process is the transformation of adenosine to inosine on a double-stranded RNA by the ADAR enzyme. In certain cases, lncRNAs are paired with these protein-coding mRNAs, which appear to anonymously codify protein genes. The double-stranded RNA region can involve ADAR enzymes that help to catalyze adenosine to inosine conversion. For example, lncRNA-PVTL5 can act as a competitive endogenous miR-126 RNA to accelerate cell proliferation by regulating the miR-126/SLC7A5 axis [43]. LncRNA PVTL5 plays a significant role in lung cancer progression. Besides, the regulatory network could be one of the molecular mechanisms for the occurrence and malignant transformation of lung cancer. Another example is the lncRNAs pseudogene, which influences gene expression at the post-transcription level. Pseudogenes are non-functional molecular relics produced in genomes that are similar to the coding gene sequence from genome family and non-functional DNA copy. For example, lncRNA PTENP1 (pseudogene PTEN) can influence the expression levels of PTEN RNA and PTEN protein by competitively binding PTEN3 and miRNA reactive elements in the UTR region [44]. The nitric oxide synthase (NOS) pseudogene (lncRNA pseudo-NOS) inhibits the translation of neuronal nitric oxide synthase (NOS) by regulating binding of ribosomes to the nitric oxide synthase (NOS) pseudogene(lncRNA pseudo-NOS) complex [45]. In conclusion, lncRNAs play important roles in human cancer development, cell cycle control, apoptosis, among other aspects. As a gene suppressor or oncogenic molecule, lncRNAs regulate gene expression transcriptionally, post-transcriptionally, and through epigenetic molecular mechanisms, such as chromatin modification, transcription, splicing, and translation. Therefore, lncRNAs regulate various physiological and pathological processes, including cell proliferation, apoptosis, heat shock response, carcinogenesis, and drug resistance. Among these functions, gene-expression can be used to elucidate how lncRNAs promote or inhibit tumorigenesis. LncRNAs have been implicated in somatic cell mutations and development of malignant tumors. LncRNAs mediate these functions by dysregulating gene expression and networks. Although the biological functions of several lncRNAs have not been established, new techniques such as Sweeping Genetic Analysis, RNA-immunoprecipitation and high-throughput sequencing, as well as gene target sequence detection and gene knockdown/knockout experiments can be used to identify the functions of lncRNAs in cancer. Besides participating in carcinogenesis, tumor invasion and metastasis, lncRNAs play a major role in tumorigenesis and the development of new regulatory factors [46]. (Figure 1C).

## Signaling pathways in lung cancer

### The Wnt/β-catenin signaling pathway

**Figure 2:**
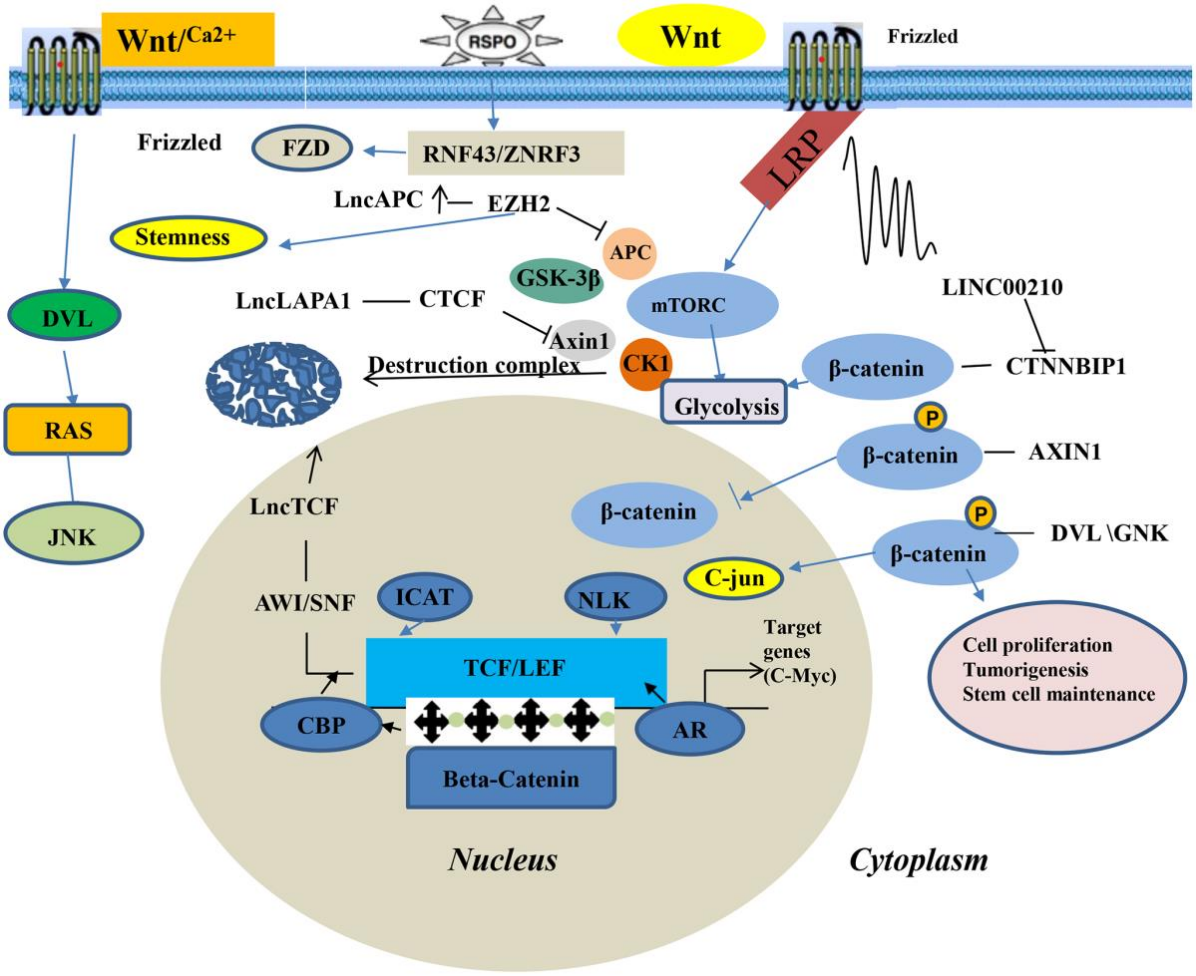
Wnt/β-catenin signaling pathway of LncRNA in lung cancer.

The Wnt signaling pathway is a complex network of protein-protein interactions that predominates embryonic and cancer development. It is also regulate biological processes of adult animals [47]. It is one of the cell signaling systems, which involves multiple aspects such as signal transduction, cell cycle, cell proliferation, apoptosis, and cell adhesion. There are three main Wnt signaling pathways: ①The Wnt/β-catenin signaling pathway is the most classical signal pathway, activated in various solid tumors, including lung cancer; ②Wnt/Ca^2+^ signaling pathway and; ③The Wnt/planar cell polarity signaling pathway (PCP signaling pathway). The function of the Wnt/β-catenin signaling pathway is important in tumor pathogenesis [48]. Studies have shown that oncogenic β-catenin is essential in regulating the occurrence and development of tumors and other physiological processes [49, 50]. In lung cancer tissues, the expression of various lncRNAs is up-regulated or down-regulated by various signaling pathways, with Wnt/β-catenin being one of the most important [51]. Xia et al. [52] documented that down-regulated MEG3 expression through the Wnt/β-catenin signaling pathway can enhance the immunity of lung cancer cell lines to cisplatin. Wei et al. [53] found that lncRNA-SVUGP2 overexpression suppressed the Wnt/β-catenin signaling pathway activity in H1975 cells. In addition, suppressed lncRNA-SVUGP2 interacts with EZH2 and activates the Wnt/β-catenin signaling pathway, promoting occurrence and development of NSCLC. It has also been shown that inhibiting miR-101-3p and activating the Wnt/β-catenin signaling pathway upregulates lncRNA SNHG1, promoting NSCLC progression. The Wnt/β-catenin signaling pathway is abnormally activated in tumor cells, which induces the β-catenin protein to enter the nucleus and interact with the T cell transcription factor/lymphoid-enhancer factor (TCF/LEF) to stimulate downstream target genes. This leads to malignant cell proliferation and tumorigenesis of malignant cells (Figure 2).

### The MAPK signaling pathway

**Figure 3:**
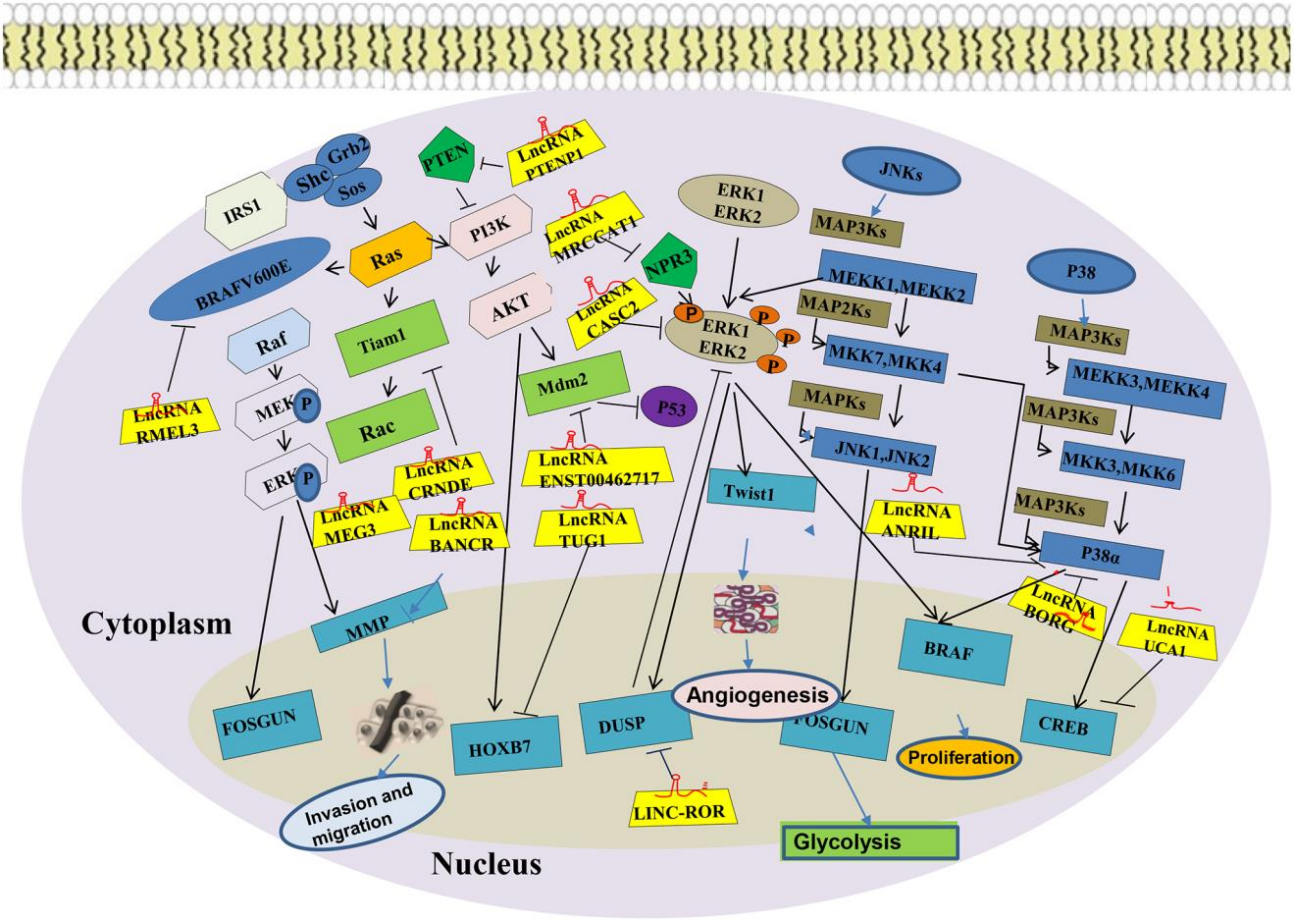
MAPK signaling pathway of LncRNA in lung cancer.

The mitogen-activated protein kinases (MAPKs) are intracellular protein kinases that transduce extracellular stimulation signals into the cell or nucleus. In so doing, they regulate numerous biochemical reactions such as cell proliferation, differentiation, apoptosis, and stress. The MAPKs are divided into extracellular c-Jun-N-terminal kinases (JNKs), extracellular signal-regulated kinases (ERKs), and P38 kinase [54]. Phosphorylation of chemicals, oxidative stress, cytokines, neurotransmitters, and other factors activate the MAPK signaling pathway. Transient excitation of ERKs promotes cell survival and proliferation, whereas their continuous excitation of ERKs promotes cell differentiation [55]. Transient excitation of JNKs promotes cell proliferation or differentiation, whereas their continuous excitation of JNKs promotes apoptosis [56]. Activation of processes downstream of proto-oncogene c-myc and nuclear factor-κB(NF-κB) by P38 induces cell death [57]. The activated MAPKs pathway enhance the occurrence of oxidative stress, inhibits the expression of the anti-apoptotic protein Bcl-2, promotes the expression of the apoptotic regulatory protein Bax, stimulates the release of cytochrome C, and finally, up-regulates cellular caspase-3,8,9 to kill the tumor cells. Upregulated expression of lncRNA TUC338 has been found to be upregulated and is associated with the prognosis of lung cancer. MAPK signaling pathway, regulated by LncRNA TUC338, is involved in of lung cancer to surrounding tissues [44]. Human TUC338 is a 590 bp gene located on chromosome12. Dysregulated TUC338 expression has also been reported in hepatocellular carcinoma and tongue squamous cell carcinoma. [58, 59]. A recent study showed that expression of MAPK correlates with drug-fast lung cancer, and some scholars have also reported that lncRNA NNT-AS1 is strongly expressed in drug-resistant NSCLC tissues and cells. This promotes NSCLC cells resistance to DDP via the MAPK/Slug signaling pathway. Ghafouri-Fard et al. [60] showed that TINCR can interact with BRAF to enhance its kinase activity, which leads to the excitation of the oncogenic mitogen-activated protein kinase (MAPK) signaling pathway, thus promoting the occurrence of NSCLC. In addition, a surgeon found that lncRNA SNHG12 up-regulates the expression of miR-181a to silence the expressions of MAPK1 and MAP2K1, and inhibits the MAPK/Slug signaling pathway by suppressing the level of phosphorylation MAPK1 (p-MAPK1), MAP2K1 (p-MAP2K1), and Slug phosphorylation [61]. SNHG12-miR-181a-MAPK/Slug axis has been established in their research, and the role of lncRNA SNHG12 in NSCLC multidrug resistance (MDR) was partially elucidated, providing a new therapeutic target for the treatment of NSCLC MDR (Figure 3).

### The STAT3 signaling pathway

**Figure 4:**
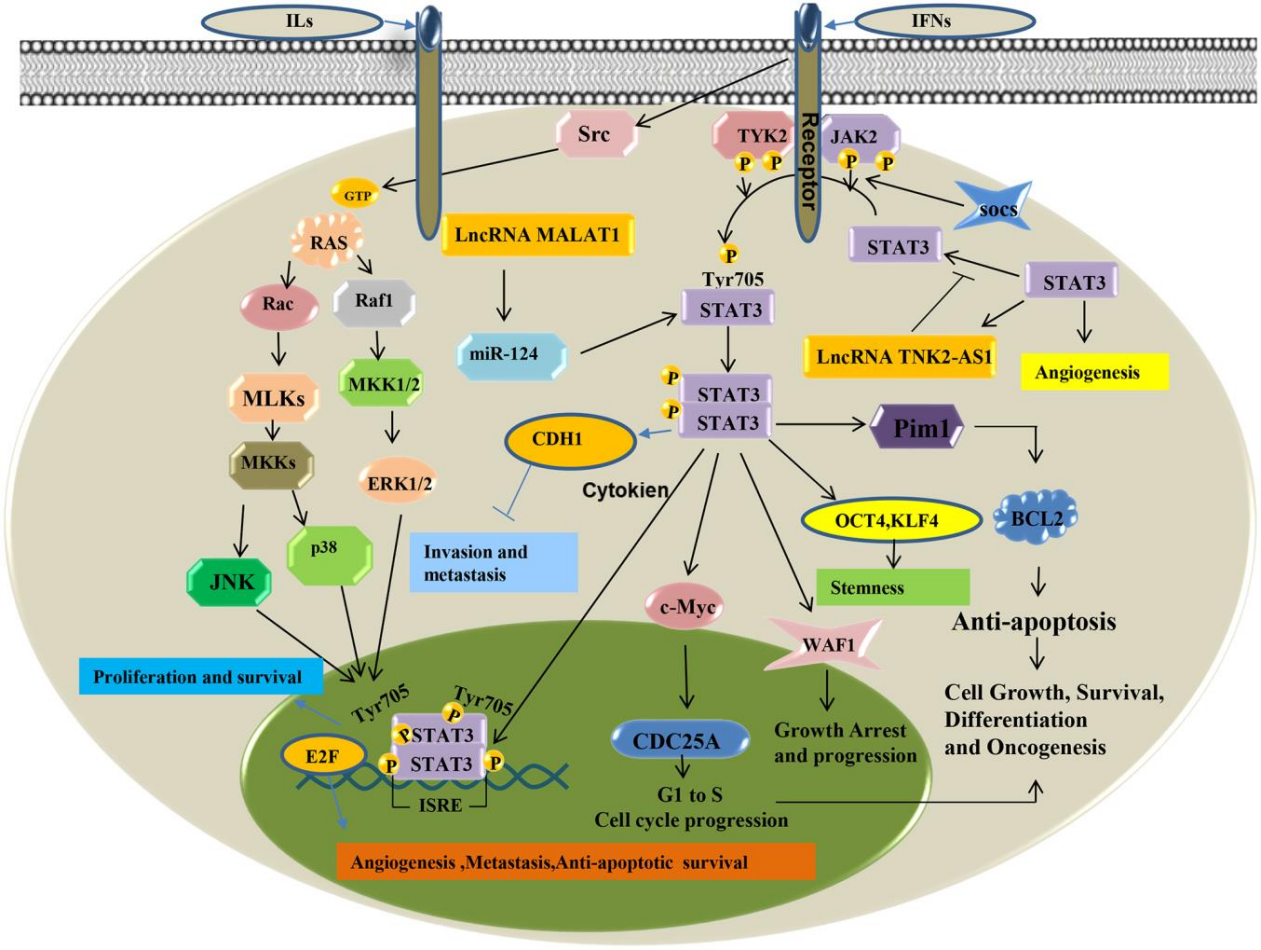
STAT3 signaling pathway of LncRNA in lung cancer.

The Signal Transducers and Activators of Transcription (STATs) related pathway is important in cytokine signal transduction. As a transcription factor, STATS3 is stimulated by cytokines such as interleukins, interferons, tumor necrosis factor, growth factors, and is activated by Janus Activated kinase (JAK), epithelial growth factor receptor (EGFR), etc. It then enters the nucleus to regulate transcription of downstream target genes [62]. Continuous activation of STAT3 is closely associated with the development of various tumors. The dysregulated epigenetic modification of STAT3 mainly consists of DNA and histone modifications. STAT3 transcription regulates epigenetic modification-related enzymes. It also regulates downstream gene expression by forming functional complexes with these enzymes. Seven mammalian members (STAT 1-4, 5a, 5b, and 6) of the STAT family were closely related in tumor progression. They are overactivated in more than 70% of human tumors and are involved in abnormal proliferation, invasion and metastasis, angiogenesis, as well immune dysregulation in tumor cells by regulating oncogenic genes. The STAT3 pathway is an important role in lung cancer. Studies have shown that lncRNA TSLNC8 significantly inhibits lung cancer cell progression and metastasis by targeting the IL-6 /STAT3/ HIF-1alpha signaling pathway. Zhao et al. [63] showed that lncRNA PICART1 induces anti-growth and anti-metastasis effects on lung cancer cells by regulating the JAK2 /STAT3 signaling pathway. While Zhang et al. [64] found that activated EGFR can upregulate the expression of PD-L1 via IL-6/JAK/STAT3 signaling pathway in non-small cell lung cancer (NSCLC) cells. The STAT3 signaling pathway may be aberrantly activated and have crucial roles in various tumor, including lung cancer (Figure 4).

### P53 signaling pathway

**Figure 5:**
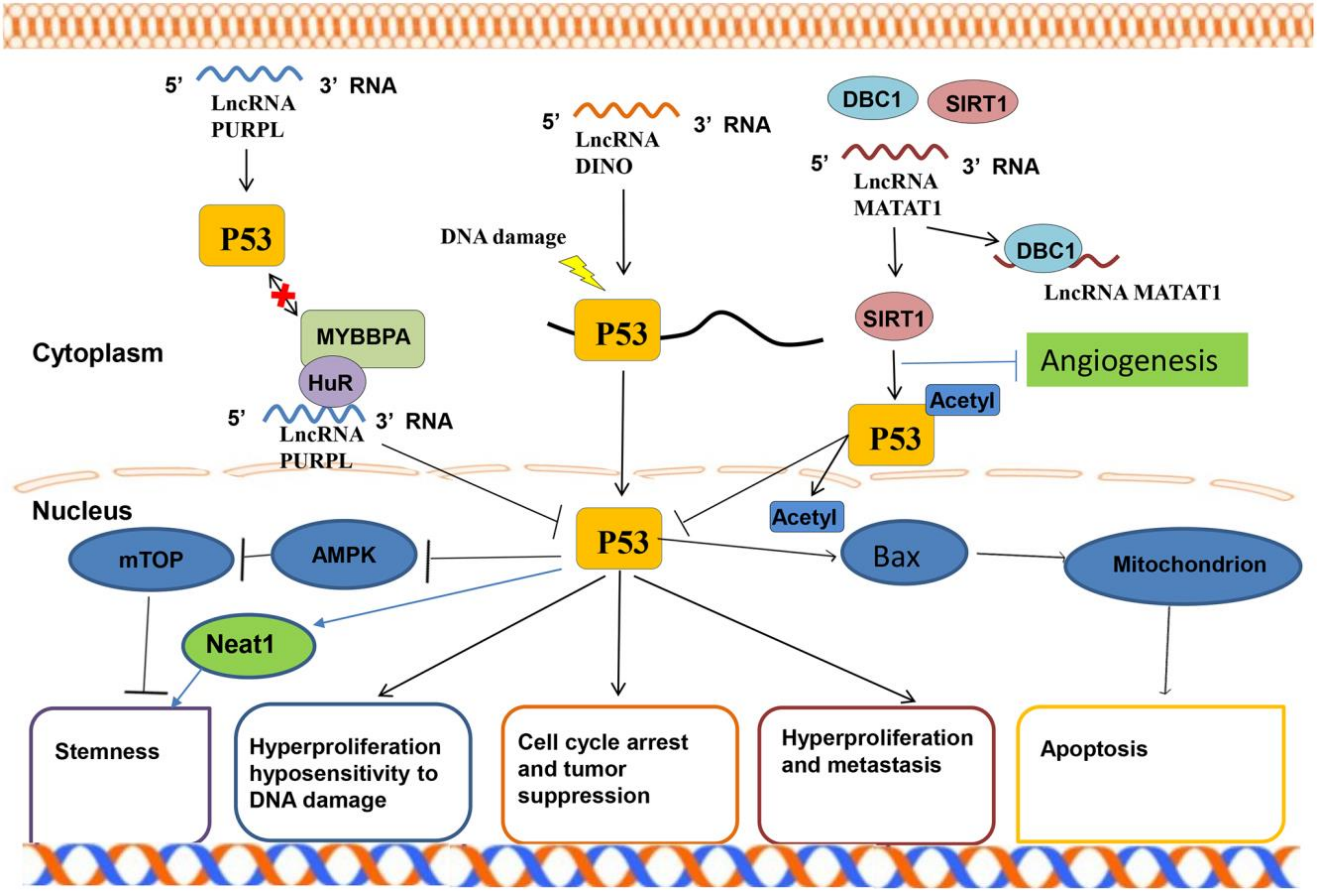
P53 signaling pathway of LncRNA in lung.

P53 is a tumor suppressor protein that regulates various genes, inhibits and promotes DNA senescence in response to genotoxicity or cellular stress reaction including apoptosis, proliferation, and cell cycle [65]. P53 is a transcription factor and is also a component of the N-terminal activation domain. P53 has a specific DNA binding center and C-terminal tetramerization domain. It has a peculiar regulatory domain that is rich in basic amino acids, and a transitory half-life, maintained by continuous ubiquitylation and subsequent degradation by the 26S proteasome at lower standards in mammalian cells. In lung cancer tissues, the expression of various lncRNAs is up-regulated or down-regulated by different signaling pathways, of which P53 is one of the important signaling pathways. Alterations in lncRNA and the signal pathway of p53 are associated with the formation of idiopathic pulmonary fibrosis and lung cancer [66]. CDKN2B-AS1 and its adjacent gene CDKN2A are down-regulated in the peripheral blood of idiopathic pulmonary fibrosis patients. This effect activates the P53 signaling pathway, which promotes the formation of lung cancer development [67]. Zhou et al. [68] found a new prognostic indicator lncRNA LOC285194 that can inhibit tumors by targeting P53 signaling pathway. Ma et al. [69] reported that knock-down lncRNA TRPM2-AS induces cell apoptosis by activating the p53-p66shc pathway, which alters the cell cycle distribution and participation in the cisplatin resistance of NSCLC cells. The P53 gene participates in several human tumors. Moreover, P53 regulates various physiological activities in cells (Figure 5).

### AKT signaling pathway

**Figure 6:**
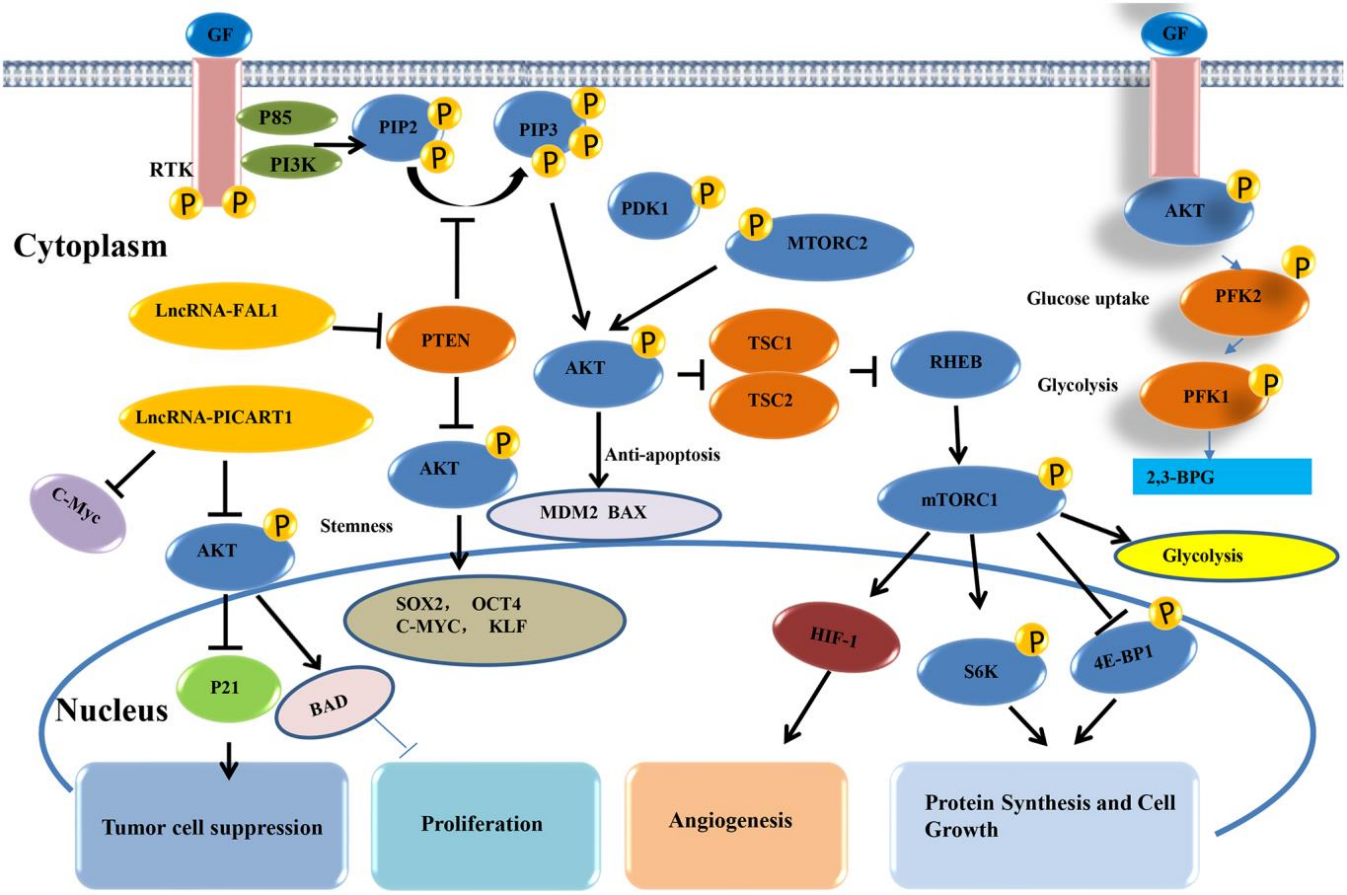
AKT signaling pathway of lncRNAs in lung cancer.

The AKT signaling pathway regulates multiple physiological processes that influence tumor occurrence and development, including metastasis, growth, proliferation, survival, transcription, and protein synthesis. In general, GF combines with receptor tyrosine kinase (RTK), then induces PIP2 to form a second messenger PIP3 through PI3K (phosphatase PTEN has the opposite effect), which activates key pathway molecules such as AKT, mTOR, and downstream gene transcriptions [70]. AKT is a proto-oncogene with important roles in regulating various cellular functions. In lung cancer, the AKT pathway is one of the major signaling pathways of lncRNAs. Dysregulation of the AKT signaling pathway contributes to the high incidences of human disease and also leads to the peak development of small molecule inhibitors of PI3K and AKT [71]. It has also been reported that lncRNA BC200 rapidly regulates the proliferation and cisplatin drug resistance of NSCLC cells through the PI3K/AKT signaling pathway [72]. Liu et al. [73] reported that the lncRNA HULC overexpression can promote cell proliferation and inhibit cell apoptosis by up-regulating the expression of SPHK1 protein expression in NSCLC, which induces the activation of its downstream PI3K/AKT signaling pathway. The cell experiments of Zhang et al. [74] revealed that lncRNA00152 inhibits the biological activities in NSCLC through the EGFR/PI3K/AKT pathway. Therefore, AKT is important in regulating various cell functions, making it an important target for human tumor diseases (Figure 6).

## Differential expression and significance of lncRNA in malignant tumors

**Figure 7:**
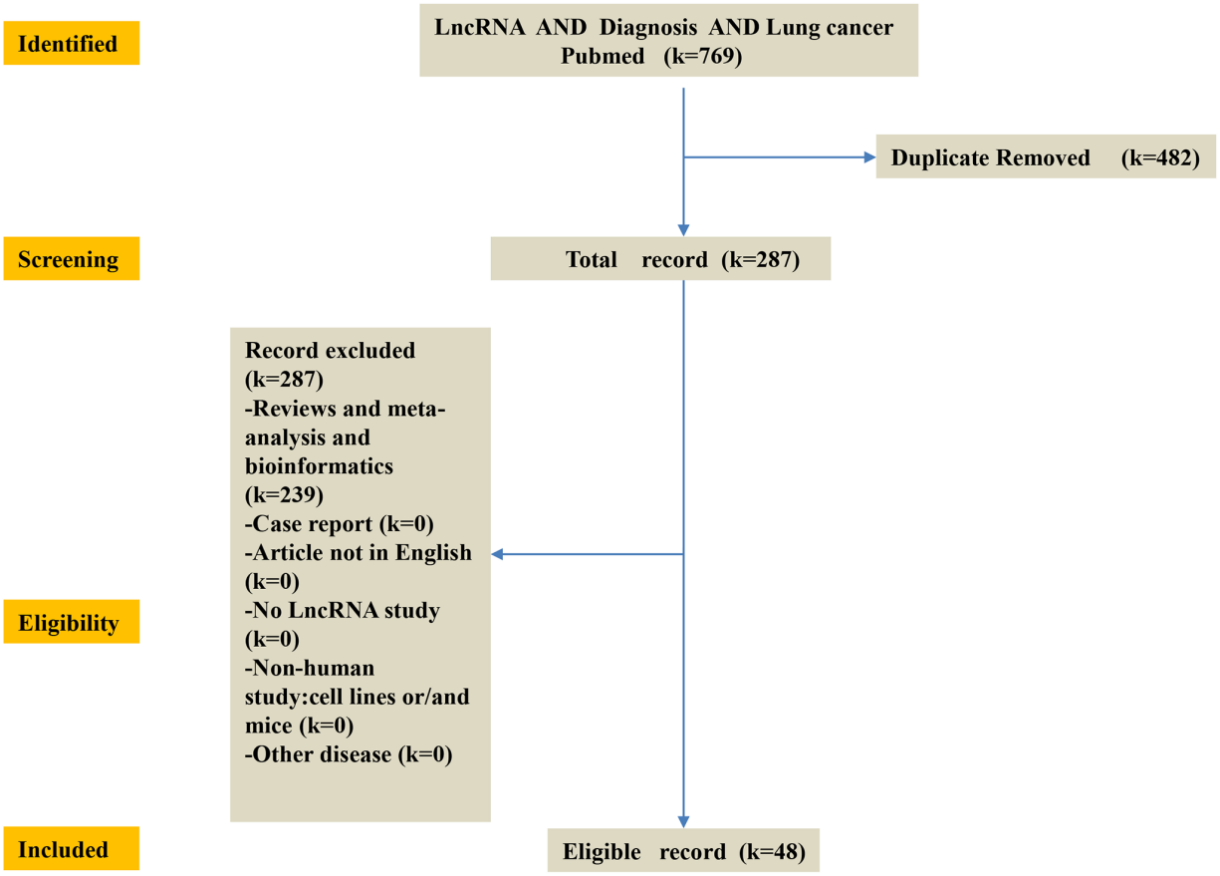
**Flow-chart of diagnosis LncRNA of this study.** K = number of literature records. First, identify the published lncRNA biomarkers of diagnosis in lung cancer patients in the NCBI PubMed database. Then, screening 769 database and obtain 287 database. The criteria of record exclusion were as follows: i) Reviews and meta-analysis and bioinformatics; ii) Case report; iii) Article not in English; iv) No lncRNA study; v) Non-human study: cell lines or/and mice; vi) Other disease. Finally, 35 up-regulated lncRNAs and 13 down-regulated lncRNAs in lung cancer were identified from these studies.

To identify the published lncRNA biomarkers for diagnosis in lung cancer patients, entry terms of “(long non-coding RNA OR lncRNA) AND (lung cancer OR LC) AND (diagnosis)” were firstly searched in the NCBI PubMed database. A total of 765 studies were obtained after removing any duplication of article content. Then we excluded some studies rigorously according to our criteria. The criteria of record exclusion were as follows: i) Reviews and meta-analysis and bioinformatics; ii) Case report; iii) Article not in English; iv) No lncRNA study; v) Non-human study: cell lines or/and mice; vi) Other tumor and disease. Finally, 35 up-regulated lncRNAs and 12 down-regulated lncRNAs in lung cancer were identified from these studies. And the exclusion criteria for other malignancies were the same as for lung cancer (Figure 7).

### LncRNAs expression and significance in malignant tumors

#### Significance of highly expressed lncRNAs in lung cancer

Globally, lung cancer is among the leading causes of morbidity and mortality. The molecular mechanism of lung cancer occurrence and metastasis is relatively complex. Thus, further studies are needed to discover more accurate lncRNAs-based diagnostic biomarkers for lung cancer. Jiang et al. [75] identified diagnosis biomarker lncRNA XLOC_009167 to predict lung cancer through the lncRNAs expression chip. In addition, lncRNA XLOC_009167 was found to be significantly up-regulated in primary lung cancer tissues and cell lines compared to para-carcinoma tissues and cell lines, and its sensitivity and specificity were higher than the conventional markers commonly used in clinical practice (CYFR21-1 and NSE). The study also showed that lncRNA XLOC_009167 can not only be used as a marker for distinguishing lung cancer patients from healthy people, and to distinguish pneumonia from early-stage lung cancer. Zhu et al. [76] found that the expression levels of lncRNA16 in lung cancer tissues are significantly elevated than in para-carcinoma tissues. Due to its high sensitivity and specificity, lncRNA16 is an appropriate plasma target for lung cancer diagnosis. Gupta et al. [77] compared the sputum of lung cancer patients with those of cancer-free smokers with benign diseases and found that three lncRNAs (SNHG1, H19, and HOTAIR) as a biomarker panel were abnormally expressed at different levels. However, the diagnostic rate of this lncRNA was low. More studies should determine whether other lncRNAs can be used as biomarkers to improve the diagnostic accuracy of lung cancer. Lin et al. [78] showed that the expression of lncRNA Small Nucleolar RNA Host Gene 1 (SNHG1) and RNA component of mitochondrial RNA processing (RMRP) in the plasma of lung cancer were higher than those of the cancer-free control group. In addition, the AUC values of lncRNA SNHG1 and RMRP were found to be 0.90 and 0.80, respectively, which could be used to distinguish between NSCLC patients from normal people. The combined diagnostic value of the combined lncRNAs was higher than that of any single lncRNA. Li et al. found that the plasma HOTAIR level in NSCLC patients was significantly higher than that of a normal person, and that the diagnostic efficiency of HOTAIR higher than that of miRNA. In addition, the composite combination of HOTAIR and CEA showed a more precise diagnosis compared to the use of HOTAIR or CEA alone. Zhang et al. [79] evaluated Distal-less homeobox 6 antisense RNA 1 (DLX6-AS1) expression levels in serum and exosomes. Compared to normal matched people, the expression of DLX6-AS1 in NSCLC tissues was higher. ROC analysis also showed that the diagnostic efficiency of DLX6-AS1 for NSCLC outperformed CYFRA21-1. Cui et al. revealed that the expression of lncRNA PVT1 significantly increased in NSCLC tissues and cell lines, with a sensitivity of 0.815 and a specificity of 0.617, and this can be used as an NSCLC diagnostic marker. Li et al. [80] also evaluated the role of P15 and P21 in PVT1-mediated proliferation via the regulation of E2F2 signaling pathway. Li et al. [81] reported that lncRNA AGAP2 antisense RNA1 (AGAP2-AS1) gene expression in NSCLC tissues was up-regulated when compared to the para-carcinoma tissue via RT-PCR. ROC curve analysis showed that AGAP2-AS1 (AGAP2 antisense RNA 1) had a potential diagnostic value for NSCLC. Wang et al. [82] discovered that the expression of lncRNA urothelial carcinoma associated 1 (UCA1) in NSCLC tissues was higher than that of paired para-carcinoma tissues. Furthermore, Receiver operating curve (ROC) analysis revealed that plasma UCA1 can be used as a diagnostic marker for NSCLC. Zhang et al. [83] found that the LINC00152 was up-regulated in lung adenocarcinoma tissues, and LINC00152 promotes lung adenocarcinoma progression. Jing et al. [84] found that both lncRNA Colon Cancer-Associated Transcript 1 (CCAT1) and SOX2 overlapping transcript (SOX2OT) can be detected in human peripheral blood cell components. Serum CCAT1 and SOX2OT levels were found to be elevated in cancer patients, and they both had certain diagnostic values. In addition, the combined application of CCAT1 and SOX2OT was more effective in the diagnosis of NSCLC than CCAT1 or SOX2OT alone. Tang et al. [85] found that three lncRNAs (RP11-397D12.4, AC007403.1, ERICH1-AS1) were up-regulated in in NSCLC patients compared to healthy individuals, and the merged area under the curve of the three lncRNAs was 0.942. More studies should however be done to ascertain their diagnostic values of NSCLC in the future. Tao et al. [86] discovered that serum exosome lncRNA TBILA and AGAP2-AS1 have a strong diagnostic effect on NSCLC. The combination of lncRNA exosomes could enhance diagnostic efficacy. Tang et al. [87] showed that LINC00342 was up-regulated in the tissues, serum, and PBMC of NSCLC patients, while the specificity and sensitivity of LINC00342 exceeded that of CYFRA21-1 for the diagnosis of NSCLC. Wan et al. [88] evaluated the expression levels and diagnostic values of PCAT6 in 349 NSCLC tissues obtained from five GEO datasets (GSE19804, GSE18842, GSE30219, GSE19188, and GSE27262). Lung cancer tissue PCAT6 exhibited the highest diagnostic value. Tan et al. [89] analyzed lncRNA X-inactive specific transcript (XIST) and HIF1A-AS1 in the serum and tumor tissues of NSCLC patients and found the levels of XIST and HIF1A-AS1 in tumor tissues or serum to exceed those of a normal person. Therefore, elevated serum XIST and HIF1A-AS1 levels can be used as predictive biomarkers for NSCLC. The positive diagnostic rate of the combination of XIST and HIF1A-AS1 exceeded that of XIST or HIF1A-AS1 alone. Wang et al. [90] reported that compared to the normal control group, lncRNA SOX2 overlapping transcripts (SOX2OT) and CCAT1 were significantly up-regulated in NSCLC serum. They combined lncRNA SOX2OT and CCAT1 to establish a diagnostic network for NSCLC that has a high efficiency. Hu et al. [91] found that during development and validation tests, circulating SPRY4-IT1 (SPRY4 Intronic Transcript 1), ANRIL, and NEAT1 levels in the plasma specimens of NSCLC patients were above normal. Receiver operating characteristic curve (ROC) results revealed that the diagnostic efficacy of ANRIL is the highest (0.798); the greater the combination of the three elements, the higher the diagnostic value (AUC: 0.876; sensitivity: 82.8%; specificity: 92.3%). Li et al. reported that the expression level of lncRNA AFAP1 Antisense RNA 1 (AFAP1-AS1) in NSCLC patients is above normal. Serum AFAP1-AS1 can be used as a molecular marker to distinguish between NSCLC patients from healthy people, with an AUC of 0.759. When AFAP1-AS1 and CyFRA21-1 were combined, their AUC was 0.860. Further analysis revealed that serum AFAP1-AS1 could be a perfect combined diagnostic biomarker for NSCLC. Luo et al. [92] evaluated the expression and clinical significance of lncRNA H19 in the plasma of NSCLC patients. Plasma H19 levels in NSCLC patients were significantly increased, and can be used as a serological marker for the subsidiary diagnosis of NSCLC. Wang et al. [93] found that lncRNA FAM83H antisense RNA 1 (FAM83H-AS1) was elevated in lung adenocarcinoma, and its expression could significantly differentiate tumors from normal lung tissues. Therefore, it can be used as a diagnostic marker. Liu et al. [94] used RT-qPCR to determine the up-regulated expression of lncRNA taurine up-regulated 1(TUG1)in LAD serum samples and cell lines, which was found to be upregulated ROC was used to evaluate the diagnostic value of TUG1 in LAD patients, and TUG1 was considered as a potential diagnostic marker for LAD (Table 1).

**Table 1:** LncRNAs are highly expressed in lung cancer.

**Order (high expression)**	**Pathology type**	**Sample**	**Method**	**Control Type**	**Cut-off value**	**AUC**	**Sensitivity (%)**	**Specificity (%)**	**PMID**
1 H19	NSCLC	Plasma	RT-PCR	BLD	6.62	0.73	67.74	63.08	29970666
2 HOTAIR	NSCLC	Plasma	qRT-PCR	HC	Unclear	0.806	76.2	71.9	28784052
HOTAIR, CEA	NSCLC	Plasma	qRT-PCR	HC	Unclear	0.841	Unclear	Unclear	28784052
3 SNHG1	LC	Plasma	ddPCR	CFC	1.11	0.9	77.78	87.88	30098474
RMRP	LC	Plasma	ddPCR	CFC	0.12	0.8	61.9	90.91	30098474
SNHG1, RMRP	LC	Plasma	ddPCR	CFC	Unclear	Unclear	84.13	87.5	30098474
4 SNHG1, H19, HOTAIR	NSCLC	Sputum	RT-PCR	CFC	0.36	0.9	82.09	89.23	31450489
	LUAD	Sputum	RT-PCR	SCC, CFC	0.36	Unclear	87.5	89.23	31450489
	LUSC	Sputum	RT-PCR	AD, CFC	0.36	Unclear	79.07	89.23	31450489
5 PVT1	NSCLC	Tissues	qRT-PCR	ANT	4.38	0.736	81.50	61.70	26490983
6 TUG1	LUAD	Cell, Serum	qRT-PCR	HC	Unclear	0.756	78.33	60.00	29254212
7 LINC00173	NSCLC	Serum	qRT-PCR	HD	0.5197	0.809	62.96	89.01	32623390
LINC00173, CEA	NSCLC	Serum	qRT-PCR	HD	Unclear	Unclear	76.85	97.8	32623390
LINC00173, Cyfra21-1	NSCLC	Serum	qRT-PCR	HD	Unclear	Unclear	82.41	95.6	32623390
LINC00173, CEA, Cyfra21-1	NSCLC	Serum	qRT-PCR	HD	Unclear	0.914	90.74	68.13	32623390
8 FEZF1-AS1	NSCLC	Plasma	qRT-PCR	HC	Unclear	0.855	67.90	85.50	32590821
FEZF1-AS1, NSE	NSCLC	Plasma	qRT-PCR	HC	Unclear	0.932	0.932	90.30	32590821
9 A panel of five lncRNAs	LUSC	Tissue	qRT-PCR	NT	Unclear	0.912	85	87	28076325
ENST00000453324	LUSC	Tissue	qRT-PCR	NT	Unclear	0.77	64	80	28076325
NR_028500	LUSC	Tissue	qRT-PCR	NT	Unclear	0.73	75	68	28076325
UC011CLY.2	LUSC	Tissue	qRT-PCR	NT	Unclear	0.8	73	80	28076325
NR_046326	LUSC	Tissue	qRT-PCR	NT	Unclear	0.77	79	71	28076325
ENST00000441841	LUSC	Tissue	qRT-PCR	NT	Unclear	0.8	86	70	28076325
10 RP11-397D12.4, AC007403.1, ERICH1-AS1	NSCLC	Plasma	qRT-PCR	CFS	8.626	0.942	93	90	26393913
11 AGAP2-AS1	NSCLC	Tissue	qRT-PCR	AT	Unclear	0.846	80	75	28617550
12 TBILA	NSCLC	Serum	qRT-PCR	HC	0.923	0.775	64.7	80.7	32015683
AGAP2-AS1	NSCLC	Serum	qRT-PCR	HC	1.12	0.734	66.7	73.3	32015683
TBILA, AGAP2-AS1	NSCLC	Serum	qRT-PCR	HC	Unclear	0.799	81.3	69.3	32015683
TBILA, AGAP2-AS1, Cyfra21-1	NSCLC	Serum	qRT-PCR	HC	Unclear	0.853	91.4	80.7	32015683
13 LINC00342, Cyfra21-1	NSCLC	Tissue, Serum, PBMC	qRT-PCR	HC	4.21	0.582	80.3	71.4	30320899
14 LINC00673	LUAD	Tissue, Plasma	qRT-PCR	NS	Unclear	0.717	unclear	unclear	28849087
15 LINC00152	NSCLC	Plasma	qRT-PCR	BD	Unclear	0.816	80	72	29375177
LINC00152, CEA	NSCLC	Plasma	qRT-PCR	HC	Unclear	0.881	76	83	29375177
16 UCA1	NSCLC	Plasma	qRT-PCR	NS	Unclear	0.912	80	88	26380024
	NSCLC	Plasma	qRT-PCR	HC	Unclear	0.886	80	88	26380024
17 LncRNA16	LC	Tissue	qRT-PCR	AT	1.945	0.858	73.97	100	27999202
18 XLOC_009167	LC	Tissue,	qRT-PCR	HC	Unclear	0.7398	78.7	61.8	30025752
	LC	Tissue	qRT-PCR	Pneumonia	Unclear	0.7005	90.1	50	30025752
19 PCAT6	LUAD	Tissue	qRT-PCR	GSE19804	Unclear	0.921	96.67	85	29238201
	LUAD	Tissue	qRT-PCR	GSE27262	Unclear	Unclear	92	96	29238201
	LUAD	Tissue	qRT-PCR	GSE30219	Unclear	Unclear	98.82	78.57	29238201
	LUAD	Tissue	qRT-PCR	GSE19188	Unclear	Unclear	86.67	90.77	29238201
	LUAD	Tissue	qRT-PCR	NC	Unclear	Unclear	95.89	87.67	29238201
	LUAD	Plasma	qRT-PCR	HD	Unclear	Unclear	87.67	97.44	29238201
	LUSC	Tissue	qRT-PCR	GSE30219	Unclear	0.9567	100	85.71	29238201
	LUSC	Tissue	qRT-PCR	GSE19188	Unclear	Unclear	96.3	92.31	29238201
	LUSC	Tissue	qRT-PCR	NC	Unclear	Unclear	100	98.04	29238201
	LUSC	Plasma	qRT-PCR	HD	Unclear	Unclear	94.12	100	29238201
20 DLX6-AS1	NSCLC	Serum	qRT-PCR	HC	Unclear	0.806	77.5	85.9	31612030
21 XIST	NSCLC	Tissue Serum	qRT-PCR	NC	Unclear	0.834	82	80	26339353
HIF1A-AS1	NSCLC	Tissue Serum	qRT-PCR	NC	Unclear	0.876	78	80	26339353
XIST, HIF1A-AS1	NSCLC	Tissue Serum	qRT-PCR	NC	Unclear	0.931	86	90	26339353
22 SOX2OT	NSCLC	Plasma	qRT-PCR	HC	Unclear	0.731	76.3	78.6	31077615
23 SOX2OT, ANRIL	NSCLC	Tissue Serum	qRT-PCR	NC	Unclear	0.853	77.1	79.2	29504701
24 SOX2OT	NSCLC	Serum	qRT-PCR	CFS	Unclear	0.846	Unclear	Unclear	31933793
CCAT1	NSCLC	Serum	qRT-PCR	CFS	Unclear	0.787	Unclear	Unclear	31933793
SOX2OT, CCAT1	NSCLC	Serum	qRT-PCR	CFS	Unclear	0.894	Unclear	Unclear	31933793
25 NEAT1	NSCLC	Tissue	qRT-PCR	AT	Unclear	0.684	80	50	25854373
26 SPRY4-IT1, ANRIL, NEAT1	NSCLC	Plasma	qRT-PCR	HC	Unclear	0.876	82.8	92.3	26453113
27 AFAP1-AS1	NSCLC	Serum	qRT-PCR	HC	Unclear	0.759	69.3	88.3	29080690
AFAP1-AS1, Cyfra21-1	NSCLC	Serum	qRT-PCR	HC	Unclear	0.86	79.3	91	29080690
28 A panel of 64 lncRNAs	NSCLC	Tissue	qRT-PCR	AT	unclear	Unclear	100	95.6	25590602
29 uc001gzl.3	LUAD	Tissue	qRT-PCR	AT	Unclear	0.719	79.4	60.3	25758555
30 CLDN10-AS1	LUAD	Tissue	MA	NT	0.078	0.847	72.03	96.3	32149133
31 ZEB2-AS1	NSCLC	Tissue	qRT-PCR	NS	Unclear	0.8793	Unclear	Unclear	32611283
32 RMRP	NSCLC	Plasma	qRT-PCR	HC	≤0.660	0.70	72.03	68.97	33425713
NEAT1	NSCLC	Plasma	qRT-PCR	HC	>1.338	0.73	67.91	68.97	33425713
TUG1	NSCLC	Plasma	qRT-PCR	HC	≤0.959	0.65	79.51	45.79	33425713
MALAT1	NSCLC	Plasma	qRT-PCR	HC	>0.507	0.66	82.86	45.74	33425713
4-lncRNA panel (RMRP, NEAT1, TUG1, and MALAT1)	NSCLC	Plasma	qRT-PCR	HC	>0.679	0.86	85.32	76.19	33425713
33 SOX2OT	LSCC	Plasma	qRT-PCR	HC	0.815	Unclear	76	73.17	30986097
34 SCAL1	LC	Blood	qRT-PCR	HC	Unclear	Unclear	Unclear	Unclear	31410144
NR-026689	LC	Blood	qRT-PCR	HC	Unclear	Unclear	Unclear	Unclear	31410144
35 LINC00487, LINC01927, LINC00959	LSCC	Tissue	Unclear	SN	0.7274	Unclear	Unclear	Unclear	31617686
LINC02315, LINC00491, LINC07049	LSCC	Tissue	Unclear	SN	0.7049	Unclear	Unclear	Unclear	31617686

#### LncRNA are highly expressed in other tumors

Highly expressed lncRNAs including SNHG1, SPRY4-IT1, H19, LINC00152, MALAT1, AFAP1-AS1, NEAT1, UCA1, HOTAIR, CCAT1, FAM83H-AS1, TUG1, ANRIL, HIF1A-AS1, PVT1 that are associated with the diagnosis of lung cancer also have a diagnostic value in other malignant tumors. Gao et al. [95] found that lncRNA SNHG1 is highly expressed in plasma and tissues from hepatocellular carcinoma (HCC) patients. Therefore, it can be used differentiate HCC patients from HCH (hepatitis B virus positive chronic hepatitis and cirrhosis) and healthy individuals. Yin et al. [96] analyzed the serum lncRNA TUG1 levels in the healthy control individuals and multiple myeloma (MM) patients and found that the serum TUG1 expression levels were up-regulated in MM patients, proving that the serum TUG1 levels is a potential diagnostic biomarker. Jing et al. [97] studied the increased expression of lncRNA SPRY4 intron transcription 1 (SPRY4-IT1) in HCC and its key role in the occurrence of liver cancer and considered it to be as a potential diagnostic indicator for liver cancer. Zhan et al. developed a urinary exosome-derived lncRNA panel (MALAT1, PCAT-1, and SPRY4-IT1) to forecast the diagnosis and relapse of bladder cancer (BC). It was found to have a great clinical value in the diagnosis and prognosis of BC. Ebru et al. [98] studied the role of plasma lncRNA H19 in the diagnosis of gastric cancer and compared it with healthy people. The level of H19 in circulation was higher than normal, showing that H19 is a potential diagnostic marker for gastric cancer. Pan et al. [99] reported that the expression of serum lncRNA H19 was up-regulated in multiple myeloma (MM), and this lncRNA could be a new biomarker for the early diagnosis and clinical treatment of MM. Zhong et al. [100] determined the diagnostic potential of lncRNA H19 for breast cancer (BC). They reported that serum exosome lncRNA H19 is a potential diagnostic biomarker for BC. Li et al. [101] found that in the plasma samples of HCC patients, HULC and LINC00152 were up-regulated in the plasma samples of HCC patients and that they have a high diagnostic value for the occurrence and metastasis of liver cancer, can be considered as a new biomarker for liver cancer. Liu et al. [102] discussed the clinical roles of LINC00152 and SNHG152 in thyroid papillary carcinoma (PTC). Notably, expressions levels of SNHG12 and LINC00152 in PTC tissues were found to be substantially higher than those in the adjacent normal tissues, and also significantly higher than those in benign thyroid nodules. ROC analysis revealed that they have a certain diagnostic value. He et al. [103] evaluated the diagnostic spectrum of lncRNA (MALAT1, AFAP1-AS1, and AL359062). From the receiver operating characteristic (ROC) curve, they found that MALAT1, AFAP1-AS1, and AL359062 can be used as new serum biomarkers for predicting the prognosis of nasopharyngeal carcinoma after diagnosis and treatment. Wen et al. [104] examined the latent value of lncRNA urothelial carcinoma associated 1 (UCA1) as a diagnostic prognostic biomarker of osteosarcoma and reported that lncRNA UCA1 is a potential specific non-invasive candidate biomarker for osteosarcoma. Liu et al. [105] documented that the plasma levels of lncRNA 91H, PVT-1, and MEG3 in CRC patients are higher than normal. A combined application can differentiate CRC patients from non-cancer control patients; and this could be a diagnostic biomarker for early CRC. Yang et al. [106] demonstrated that serum PVT1 levels in cervical cancer tissues was higher than in adjacent normal tissues, forming it important diagnostic marker. Sun et al. [107] found that four lncRNA (HOTAIR, PVT1, XLOC_000303, and AL592284.1) were up-regulated in legacy cancer (CC) compared to the cervical cancer control group. The risk scoring formula was used to analyze ROC, and it was found that a combination of the four factors have a higher diagnostic value. Chen et al. [108] evaluated the published lncRNA PVT1 expression data and found that PVT1 was up-regulated in melanoma tissues compared with para-carcinoma tissues. Serum PVT1 levels were significantly higher in melanoma patients compared with age and sex-matched nonmelanoma and melanocytic nevus controls. The ROC analysis showed that the serum PVT1 level can distinguish the melanoma patients and the control group. Luo et al. [109] selected 6 lncRNAs can be used for the diagnosis of HCC based on RT-qPCR. The level of plasma lncRNA ZFAS1 in HCC was substantially higher than the average level. The results indicated that ZFAS1 can be used as a marker for HCC diagnosis (Table 1).

### LncRNA expression is suppressed in malignant tumors

#### LncRNA expression is suppressed in lung cancer

Compared with the lncRNAs with high expression in lung cancer, the number of lncRNAs with low expression in lung cancer is relatively small. Wang et al. [110] found that the expression levels of lncRNA (C6orf176-TV1 and C6orf176-TV2) in NSCLC tissues were significantly lower than those in paired adjacent tissues. Then, they also found that C6orf176 can be applied in the clinical diagnosis of NSCLC. Liang et al. [111] found that the plasma lncRNA Growth Arrest Specific transcript 5 (GAS5) levels in NSCLC patients were down-regulated. They further found that a combination of GAS5 and CEA provided a more accurate diagnosis for NSCLC patients. Kamel et al. [112] reported that, compared to CEA, lncRNA GAS5 and SOX2OT expression are more sensitive and specific NSCLC biomarkers. Moreover, their diagnostic sensitivity and specificity when combined are higher than that of GAS5 and SOX2OT used alone. Hao et al. [113]. found that lncRNA MAG12 antisense RNA 3 (MAG12-AS3) in NSCLC were significantly down-regulated compared to normal controls. Zeng et al. [114] found that knockdown of lncRNA ZNFX1 antisense RNA1 (ZFAS1) can suppress NSCLC cell proliferation, as well as invasive potentials, increased NSCLC cell apoptotic rates *in vitro*. Tan et al. [115] found that LINC00312 was significantly down-regulated in NSCLC tissues compared to normal tissues. They further evaluated the significance of LINC00312 in NSCLC subtypes such as ADC and SCC using the ROC curve. The results revealed that LINC00312 can effectively identify NSCLC. Wang et al. [116] reported that lncRNA PRAL levels in most NSCLC tumor tissues are significantly lower than those in para-carcinoma and healthy tissues. ROC curve analysis showed that there is a strong difference between NSCLC and the control group, implying that PRAL expression levels can be used as a latent biomarker for diagnosis for NSCLC diagnosis. Guo et al. [117] evaluated the expression levels of lncRNA MALAT1 in the whole blood of NSCLC and found that they were low compared to normal controls, while the AUC was 0.718. Further results showed that MALAT1 can be used as a predictive biomarker of lung cancer. Weber et al. [118] also evaluated the effectiveness of lncRNA MALAT1 as a blood marker for NSCLC. They found that MALAT1 was not sensitive enough to be used as a single biomarker for the diagnosis of the NSCLC in the blood, but could be used as an auxiliary biomarker within a group to improve the overall diagnostic performance. Chen et al. [119] found that the plasma lncRNA RP11-438N5.3 levels in NSCLC patients were low than normal. ROC analysis showed that lncRNA RP11-438N5.3 could be a new biomarker for NSCLC. Wang et al. [120] reported many significant differences in the expressions of lncRNA and mRNA expression in early lung adenocarcinoma and the corresponding adjacent nontumorous tissues (NT). They later established a lncRNA prediction panel (ENST00000540136, NR_034174, uc001gzl. 3, uc004bbl. 1, ENST00000434223). Further validation showed that these lncRNAs had a high sensitivity and specificity in the identification of early lung adenocarcinoma and NT samples. Jin et al. [121] found that CLDN10-AS1 was up-regulated in lung adenocarcinoma tissues (LUAD) from the GEO database, while SFTA1P, SRGAP3-AS2 and ADAMTS9-AS2 were down-regulated. According to the sensitivity and specificity indices and ROC curve, lncRNA SFTA1P, ADAMTS9-AS2, CLDN10-AS1 (up-regulated) and SRGAP3-AS2 all have a diagnostic value for LUAD. Currently, lncRNAs that have been found to be suppressed have previously not been shown to be viable diagnostic markers for lung squamous cell carcinoma. In this study, we found that suppressed lncRNAs have a certain significance in lung squamous cell carcinoma, and their potential as diagnostic markers should be evaluated. Li et al. [122] reported that lncRNA SFTA1P (Surfactant associated with 1, pseudogene) is significantly down-regulated in LSCC tissues compared to para-carcinoma tissues. In addition, HNRNP-U (heterogeneous nuclear ribonucleoprotein U) expression was shown to be down-regulated in LSCC, and was positively associated with poor prognosis as well as SFTA1P. Li et al. [123] evaluated biomarkers of smoking- related lung squamous cell carcinoma (LSCC) and found that LINC00094 was down-regulated in tumor tissues of smoking patients, suggesting that LINC00094 could be a biomarker or therapeutic target for smoking-related LSCC. He et al. [124] showed that MAGI2-AS3 is significantly down-regulated in the tumor tissues in lung squamous cell carcinoma (LSCC) compared to para-carcinoma tissue. Further analysis revealed that MAGI2-AS3 inhibits LSCC by regulating the miR374a/b-5p /CADM2 axis, hence it may a potential biomarker of LSCC. Xiong et al. [125] found that lncRNA FEZF1 antisense RNA 1(FEZF1-AS1) in lung squamous cell carcinoma (LSCC) tissues was significantly lower than that in the para-carcinoma tissues; and its low expression was correlated with a poor prognosis of LSCC tissues, indicating that FEZF1-AS1 could be a specific biomarker for LSCC. Studies have revealed that the above lncRNAs are down-regulated in lung squamous cell carcinoma. Table 2).

**Table 2:** LncRNAs are lowly expressed in lung cancer.

**Order (Low expression)**	**Pathology type**	**Sample**	**Method**	**Control Type**	**Cut-off value**	**AUC**	**Sensitivity (%)**	**Specificity (%)**	**PMID**
1 C6orf176-TV1	NSCLC	Tissue	qRT-PCR	PLT	24.239	0.708	51	88	27374438
C6orf176-TV2	NSCLC	Tissue	qRT-PCR	PLT	25.646	0.64	49	75	27374438
2 GAS5	NSCLC	Plasma	qRT-PCR	HC	Unclear	0.832	82.2	72.7	27631209
GAS5, CEA	NSCLC	Plasma	qRT-PCR	HC	Unclear	0.909	86.7	90.9	27631209
3 GAS5	NSCLC	Plasma	qRT-PCR	HC	Unclear	0.814	82.5	80	31077615
4 GAS5	NSCLC	Tissue	qRT-PCR	PLT	Unclear	0.653	60	70	28339045
	NSCLC	Plasma	qRT-PCR	HC	Unclear	0.638	42.31	77.78	28339045
5 MAGI2-AS3	LUAD	Plasma	qRT-PCR	HC	Unclear	0.866	82.4	85	29922089
	LUSC	Plasma	qRT-PCR	HC	Unclear	0.887	90.9	85	29922089
	LUAD	Platelets	qRT-PCR	HC	Unclear	0.853	77.9	81.7	29922089
	LUSC	Platelets	qRT-PCR	HC	Unclear	0.892	78.7	88.3	29922089
	LUAD	Plasma	qRT-PCR	HC	Unclear	0.806	75	73.3	29922089
ZFAS1	LUSC	Plasma	qRT-PCR	HC	Unclear	0.77	66.7	80	29922089
	LUAD	Platelets	qRT-PCR	HC	Unclear	0.78	75	66.7	29922089
	LUSC	Platelets	qRT-PCR	HC	Unclear	0.744	93.9	43.3	29922089
MAGI2-AS3, ZFAS1	LUAD	Plasma	qRT-PCR	HC	Unclear	0.89	76.5	86.7	29922089
	LUSC	Plasma	qRT-PCR	HC	Unclear	0.902	84.9	88.3	29922089
	LUAD	Platelets	qRT-PCR	HC	Unclear	0.908	89.7	86.7	29922089
	LSCC	Platelets	qRT-PCR	HC	Unclear	0.919	81.8	96.7	29922089
6 LINC00312	NSCLC	Tissue	qRT-PCR	NT	Unclear	0.803	Unclear	Unclear	28849087
7 PRAL	NSCLC	Tissue	qRT-PCR	NT	Unclear	0.8546	Unclear	Unclear	30274002
8 MALAT-1	NSCLC	Plasma	qRT-PCR	CFC	–0.41	0.79	56	96	24313945
	LUAD	Plasma	qRT-PCR	CFC	–1.44	0.75	81	64	24313945
	LUSC	Plasma	qRT-PCR	CFC	–0.41	0.82	63	96	24313945
9 MALAT-1	LC	Blood	qRT-PCR	HC	10.344	0.718	70	60	26137228
10 RP11-438N5.3	NSCLC	Plasma	qRT-PCR	HC	Unclear	0.814	Unclear	Unclear	32184656
11 ENST00000434223	LUAD	Tissue	qRT-PCR	PLT	Unclear	0.843	81	79.4	25758555
uc004bbl.1	LUAD	Tissue	qRT-PCR	PLT	Unclear	0.821	85.2	62.4	25758555
ENST00000540136	LUAD	Tissue	qRT-PCR	PLT	Unclear	0.882	79.4	84.1	25758555
NR 034174	LUAD	Tissue	qRT-PCR	PLT	Unclear	0.835	77.8	79.4	25758555
12 SFTAP1	LUAD	Tissue	MA	NT	39.89	0.9126	82.29	100	32149133
ADAMTS9-AS2	LUAD	Tissue	MA	NT	0.212	0.9116	81.49	96.3	32149133
SRGAP3-AS2	LUAD	Tissue	MA	NT	0.921	0.7945	73.04	75.93	32149133
13 Exo-GAS5	NSCLC	Serum	qRT-PCR	HC	Unclear	0.857	85.94	70	31032916
CEA+Exo-GAS5	NSCLC	Serum	qRT-PCR	HC	Unclear	0.929	89.06	90	31032916

#### LncRNA is down-regulated in malignant tumors

LncRNAs such as AFAP1-AS1, ADAMTSP-AS2, and GAS5 with suppressed expression levels in lung cancer are rarely used as diagnostic markers for other malignant tumors. Esfandi et al. [126] evaluated the clinical value of lncRNA AFAP1-AS1 in gastric cancer and confirmed that AFAP1-AS1 is down-regulated in gastric cancer compared to para-carcinoma tissues, and could therefore, be used as a diagnostic biomarker. Chen et al. [127] evaluated the expression and diagnostic significance of the two new ceRNAs in esophageal squamous cell carcinoma (ESCC). CADM2 and ADAMTS9-AS2 were under-expressed in ESCC compared to para-carcinoma tissues. Du et al. [128] evaluated the diagnostic efficacy of urine lncRNAs for bladder cancer (BC). They constructed a two-lncRNA panel (UC004COX.4, GAS5), and the area under the curve showed that the two-lncRNA panel had a high BC diagnostic accuracy. Permuth et al. [129] developed a NanoString nCounter^®^ technology for the diagnosis of intraductal papillary mucinous neoplasms (IPMNs) cases for cystic pancreatic ductal adenocarcinoma (PDAC) precursors. They found that lncRNA GAS5 expression was suppressed when compared to the healthy control group, and that two lncRNAs (GAS5 (low expression) and SRA (high expression) could distinguish IPMN cases from the non-diseased control group (Table 2).

## Future outlook

Early screening and diagnosis were an effective way for enhancing survival outcomes for patients with malignant tumors. Studies have shown that, when compared to non-tumor tissues many lncRNAs are significantly up-regulated or down-regulated in tumor tissues compared with non-tumor tissues, and their differences in expression have a clinical diagnostic value. Novel diagnostic lncRNAs play a significant role in the early screening and of malignant tumors, which improves prognosis. Most studies have combined lncRNAs with micro-RNA and other tumor markers to improve their diagnostic performance. However, more comprehensive studies are still needed to clarify the diagnostic value of lncRNAs in cancers. Studies have proposed various clinical applications for these molecules. Even though some challenges are yet to be solved, lncRNAs play important roles in tumor development [130]. Large-scale and high-quality studies are needed to validate their clinical applications as cancer biomarkers.

### Data availability statement

All data generated or analyzed during this study are included in this article.
